# Stellenwert des 1,5-T-MR-Linearbeschleunigers für die primäre Therapie des Prostatakarzinoms

**DOI:** 10.1007/s00117-021-00882-8

**Published:** 2021-07-23

**Authors:** Daniel Wegener, Daniel Zips, Cihan Gani, Simon Boeke, Konstantin Nikolaou, Ahmed E. Othman, Haidara Almansour, Frank Paulsen, Arndt-Christian Müller

**Affiliations:** 1 Universitätsklinik für Radioonkologie, Universitätsklinikum Tübingen, Eberhard Karls Universität Tübingen, Hoppe-Seyler-Str. 3, 72076 Tübingen, Deutschland; 2 Universitätsklinik für Radiologie, Eberhard Karls Universität Tübingen, Tübingen, Deutschland; 3 Universitätsklink für Neuroradiologie, Johannes Gutenberg-Universität Mainz, Mainz, Deutschland; 4 Klinik für Radioonkologie und Strahlentherapie, Ludwigsburg, Deutschland

**Keywords:** Adaptive Strahlentherapie, MR-geführte Radiotherapie, Linearbeschleuniger, Weichteilkontrast, Lagerungskontrolle, Adaptive radiotherapy, MR-guided radiotherapy, Radiosurgery, Soft tissue contrast, Patient positioning

## Abstract

**Hintergrund:**

Der potenzielle Nutzen des verbesserten Weichteilkontrastes von MR-Sequenzen gegenüber der Computertomographie (CT) für die Radiotherapie des Prostatakarzinoms ist bekannt und führt zu konsistenteren und kleineren Zielvolumina sowie verbesserter Risikoorganschonung. Hybridgeräte aus Magnetresonanztomographie (MRT) und Linearbeschleuniger (MR-Linac) stellen eine neue vielversprechende Erweiterung der radioonkologischen Therapieoptionen dar.

**Material und Methoden:**

Dieser Artikel gibt eine Übersicht über bisherige Erfahrungen, Indikationen, Vorteile und Herausforderungen für die Radiotherapie des primären Prostatakarzinoms mit dem 1,5-T-MR-Linac.

**Ergebnisse:**

Alle strahlentherapeutischen Therapieindikationen für das primäre Prostatakarzinom können mit dem 1,5-T-MR-Linac abgedeckt werden. Die potenziellen Vorteile umfassen die tägliche MR-basierte Lagekontrolle in Bestrahlungsposition und die Möglichkeit der täglichen Echtzeitanpassung des Bestrahlungsplans an die aktuelle Anatomie der Beckenorgane (adaptive Strahlentherapie). Zusätzlich werden am 1,5-T-MR-Linac funktionelle MRT-Sequenzen für individuelles Response-Assessment für die Therapieanpassung untersucht. Dadurch soll das therapeutische Fenster weiter optimiert werden. Herausforderungen stellen u. a. die technische Komplexität und die Dauer der Behandlungssitzung dar.

**Schlussfolgerung:**

Der 1,5-T-MR-Linac erweitert das radioonkologische Spektrum in der Therapie des Prostatakarzinoms und bietet Vorteile durch tagesaktuelle MRT-basierte Zielvolumendefinition und Planadaptation. Weitere klinische Untersuchungen sind notwendig, um die Patienten zu identifizieren, die von der Behandlung am MR-Linac gegenüber anderen strahlentherapeutischen Methoden besonders profitieren.

## Abstract (en)

**Background:**

Hybrid devices of MR-scanners and linear accelerators (MR-Linacs) represent a new and promising extension of radiotherapeutic options for prostate cancer. The potential advantage of magnetic resonance imaging (MRI) over computed tomography (CT) for soft tissue contrast is well-known and leads to more consistent and smaller target volumes and improved normal tissue sparing.

**Objectives:**

This article presents an overview of clinical experience, indications, advantages and challenges of utilizing a 1.5 T MR-Linac in the setting of radiotherapy of prostate cancer.

**Results:**

All current indications for radiotherapy of prostate cancer can be treated with an MR-Linac. The advantages include daily MR-based imaging in treatment position and daily adaption of the treatment plan on current anatomy (adaptive radiotherapy). Additionally, functional MRI sequences might be exploited to enhance treatment individualization and response assessment. Ultimately treatment on an MR-Linac might further increase the therapeutic window. The limitations of using MR-Linac include treatment complexity and the duration of each session.

**Conclusions:**

MR-Linacs expand the spectrum of radiotherapeutic options for prostate cancer. Increased precision can be reached with daily MRI-based target volume definition and plan adaption. Clinical studies are necessary to identify patient groups who would benefit most from radiotherapy on a MR-Linac.

## Status quo

### Indikation

Die perkutane Strahlentherapie bietet für lokalisierte Prostatakarzinome eine kurative Therapieoption als onkologisch gleichwertige Alternative zur radikalen Prostatektomie [1]. Patienten werden im interdisziplinären Konsens ausführlich über die Vor- und Nachteile der verschiedenen kurativen Therapieoptionen je nach Risikokonstellation (aktive Überwachung, Strahlentherapie, Operation) informiert.

### Standard der Bestrahlungstechnik

Aktueller Standard ist eine intensitätsmodulierte Radiotherapie (IMRT), häufig als „volumetric arc therapy“ (VMAT) mit täglicher Positionskontrolle und -anpassung auf Basis täglicher „on-board“ cone-beam CTs (cbCT) an den Bestrahlungsplan [1]. Dabei wird die Tischposition täglich korrigiert, um den initial angefertigten Bestrahlungsplan bestmöglich reproduzieren zu können.

### Strahlentherapie-Planung

Standardmäßig erfolgt die CT-gestützte Planung einer IMRT der Prostata heutzutage unter Zuhilfenahme einer diagnostischen Magnetresonanztomographie (MRT) der Prostata oder besser einer (anatomisch exakten) Planungs-MRT in Bestrahlungsposition. Es ist bereits seit Jahren bekannt, dass eine MRT-basierte Definition des Zielvolumens und der Risikoorgane zu signifikant kleineren Zielvolumina führt und dadurch eine geringere Intra- und Interobserver-Variabilität erreicht wird [2]. Hierdurch kann die Strahlenbelastung der Risikoorgane und damit die Nebenwirkungsrate reduziert werden [2].

### Positionierungskontrolle

**Figure 1:**
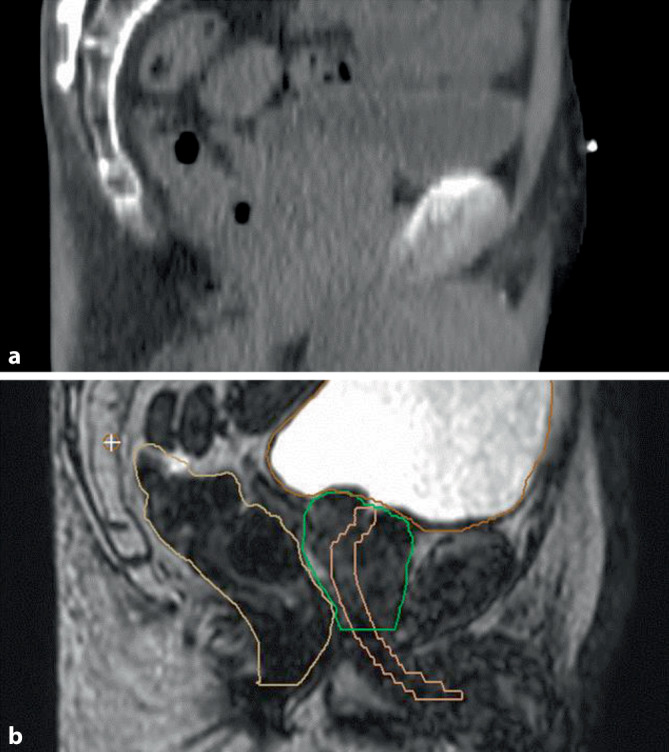

Bisher war während der Bestrahlungsserie die tägliche „on-board“-Positionskontrolle auf (native) kV- oder MV-CT-Bilder beschränkt. Der Weichteilkontrast von Prostata zu Rektumvorderwand, Blase und weiteren umliegenden Strukturen ist dabei eingeschränkt (Abb. 1; [3]). Die Bestrahlungsposition wird in der klinischen Routine anhand der CT-Anatomie der Beckenorgane, knöcherner Hilfsstrukturen und implantierter Goldmarker festgelegt und ggf. korrigiert [4]. Für die intrafraktionelle Positionskontrolle stehen kV-, MV-Röntgenaufnahmen, ultraschallbasierte und Oberflächendetektionstechniken zur Verfügung.

### Hypofraktionierung, Ultrahypofraktionierung und intraprostatischer Boost

Die zunehmende Evidenz und Verbreitung von moderater sowie extremer Hypofraktionierung bzw. Ultrahypofraktionierung, z. B. 7 × 6,1 Gy oder 5 × 7,25 Gy [5, 6] sowie die Verwendung eines Boosts auf intraprostatische Tumorläsionen [7] erweitern in Zukunft das Therapiespektrum der modernen Radioonkologie. Eine wichtige Herausforderung für den sicheren Einsatz ultrahypofraktionierter Konzepte ist die inter- und intrafraktionelle Variabilität des Zielvolumens (Prostata ± Samenblasen) und der angrenzenden Risikoorgane (v. a. Rektumvorderwand, Harnblase und Urethra) von jeweils mehreren Millimetern insbesondere in anteroposteriorer Dimension [8, 9]. Dabei sind Deformierungen des Zielvolumens ebenfalls möglich [8]. Die Sicherstellung der punktgenauen Dosisapplikation stellt eine Limitation der bisherigen Radiotherapieplanung und Durchführung dar [3].

## Konzept des MR-Linac

Äußerst vielversprechend, insbesondere für die primäre Radiotherapie des Prostatakarzinoms, ist bezüglich der o. g. Einschränkungen das Hybridgerät aus MRT und Linearbeschleuniger (MR-Linac). Ziel dieser Technologie ist es, tägliche anatomische Kontrollen *online* anhand anatomischer MRT-Sequenzen zu ermöglichen und zusätzlich tagesindividuelle Adaptationen der Bestrahlungspläne anhand der aktuellen Anatomie zu ermöglichen. Letztlich sollen so die Heilungsraten weiter verbessert und Nebenwirkungen reduziert werden [10].

**Figure 2:**
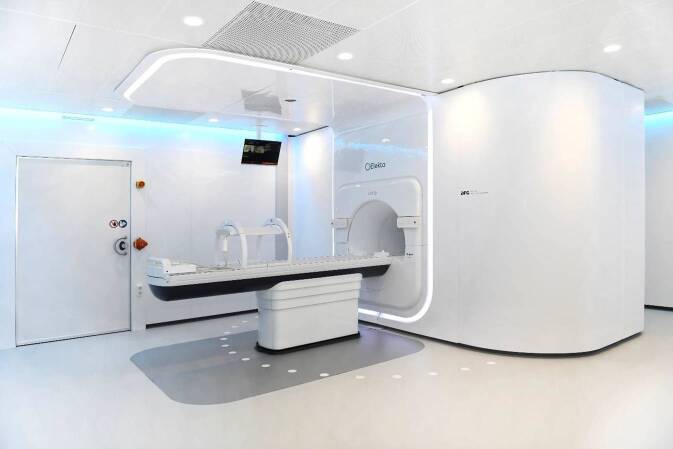

Aktuell werden MR-Linacs von zwei Firmen klinisch vertrieben: Der 1,5 T Unity^TM^ (Elekta AB, Stockholm, Schweden) und der 0,35 T MRIdian^TM^ (Viewray Inc, Oakwood, Ohio, USA). Bezüglich des Aufbaus, Workflow, technischer Spezifikationen und Limitationen sei auf weiterführende Literatur verwiesen [11–14]. Einen hervorragenden, aktuellen Überblick gibt der Artikel von Hoegen et al. [10]. In Tübingen wird ein 1,5-T-MR-Linac seit 2018 klinisch betrieben (Abb. 2).

### Kooperation zwischen Radiologie und Radioonkologie

Dieses neue Hybridgerät stellt für Radioonkologen einen Paradigmenwechsel dar und setzt einerseits neue Arbeitsabläufe voraus, insbesondere das tägliche *Live*-Adaptieren von Bestrahlungsplänen. Andererseits rückt nun in großem Ausmaß die komplexe MR-Technologie und -Bildakquisition und insbesondere deren Bewertung in den Vordergrund. Radioonkologen, Medizinphysiker und MTRA der Strahlentherapie benötigen eine Ausbildung in der MR-Diagnostik und auch Grundlagenwissen für die Anwendung der MRT in der Strahlentherapie. Aufgrund eigener Erfahrungen erachten es die Autoren als unverzichtbar, dass bereits vor der klinischen Einführung eine enge Kooperation mit der diagnostischen Radiologie und MRT-erfahrenen Medizinphysikern stattfindet. Zusätzlich haben sie eine DFG-geförderte wissenschaftliche Kooperation zwischen Radiologie, MR-Physik, biomedizinischer Physik und Radioonkologie etabliert, um die MR-geführte Strahlentherapie als innovative Form der Präzisionsmedizin weiterzuentwickeln und den klinischen Stellenwert dieser Technologie zu validieren.

## Therapie des Prostatakarzinoms am 1,5-T-MR-Linac

Im Folgenden werden bisherige Studienergebnisse, Erfahrungen, Chancen und Herausforderungen insbesondere des 1,5-T-MR-Linac (MR-Linac) für die Therapie des primären Prostatakarzinoms dargestellt.

### Machbarkeit

Die primäre Radiotherapie des lokalisierten Prostatakarzinoms kann am MR-Linac sicher durchgeführt werden [15–17]. Alle aktuellen Konzepte können sehr gut realisiert werden. Dabei wird vor jeder Bestrahlung eine schnelle anatomisch optimierte Sequenz angefertigt (T2w oder T2*w) und die Beckenanatomie evaluiert sowie der Behandlungsplan entsprechend adaptiert. In ersten Studien konnte für das Clinical Target Volume (CTV) am MR-Linac eine geringe Dosisvarianz im Behandlungsverlauf gezeigt werden ([18]; CTV D99 % Abdeckung −2,2 % ± 2,9 %), was einer konstant guten Abdeckung des Zielvolumens bei jeder Fraktion entspricht. Dadurch blieb gleichzeitig auch die Belastung der Risikoorgane konstant gering (Dosisvarianz an Harnblase +1,6 % ± 2,3 %, am Rektum −0,2 % ± 2,2 %, bezogen jeweils auf dasjenige Volumen, welches 60 Gy oder mehr erhielt).

Vor allem für diejenigen Patienten, welche größere interfraktionelle Organbeweglichkeit im Becken zeigen, konnte ein dosimetrischer Vorteil der Bestrahlung am MR-Linac gegenüber einem *normalen* Linac gezeigt werden. Andererseits spielt für diejenigen Patienten, welche über die Dauer der Radiotherapie eine stabile Anatomie präsentieren, der Vorteil der täglichen Adaptation am MR-Linac keine entscheidende Rolle [15].

### Visualisierung

Für die Bestrahlungsplanung anhand T2w und T2*w konnte am MR-Linac im Vergleich zur CT eine deutlich reduzierte Intra- und Interobserver-Variabilität gezeigt werden [19].

**Figure 3:**
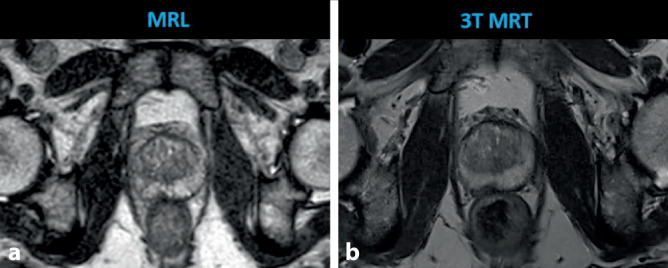

Die Bildqualität am MR-Linac ist aufgrund der technischen Eigenschaften (z. B. nur 8‑Kanal-Empfängerspule, geteilter Magnet des Hybridgeräts) gegenüber diagnostischen MRT-Scannern eingeschränkt (Abb. 3; [20]). Hervorzuheben ist, dass die MR-Bildgebung am MR-Linac nicht zu diagnostischen Zwecken erfolgt, sondern zur Bildführung der adaptiven Bestrahlung dient. Wichtig sind dafür anatomisch exakte, schnelle Sequenzen, um jede Bestrahlung präzise, rasch und ohne Positionsveränderung durch Organfüllung oder Patientenbewegung durchführen zu können [21]. Die Bildqualität von T2w- und diffusionsgewichteter Bildgebung (DWI) einer diagnostischen 3‑T-MRT wurde mit *Routine*-Sequenzen des 1,5-T-MR-Linac in einer prospektiven Studie verglichen. Darin konnte gezeigt werden, dass eine annährend vergleichbare Qualität einer schnellen 2‑minütigen T2w-Sequenz am MR-Linac im Vergleich zu einer diagnostischen 3‑T-MRT-T2w-Sequenz (Letzteres mit Spasmolytikum) erzielt werden kann [20]. Auch für einen Boost intraprostatischer Tumorläsionen erscheint der 1,5-T-MR-Linac aufgrund des MRT-Weichteilkontrasts gut geeignet [22].

### Intrafraktionelle Lageverschiebung und Positionierungskontrolle

Aktuell dauert eine Bestrahlungssitzung am 1,5-T-MR-Linac pro Patient 20–45 min („in-room time“, Durchschnitt ca. 30 min; zum Vergleich: an einem Linac liegt die tägliche Dauer bei ca. 10 min, [23]). Dabei erhöht sich das Risiko für eine intrafraktionelle Lageverschiebung der Beckenorgane mit der Liegedauer und ist prinzipiell am MR-Linac größer als an einem *normalen* Linac [21]. Die Lagekontrolle und -korrektur während der gesamten Prozedur ist daher besonders wichtig. Dies wird durch mehrfache Kontrollen während der adaptiven Planung und durch „motion monitoring“ während der Strahlapplikation sichergestellt, d. h. die MRT-Bildgebung erfolgt zeitgleich während der Bestrahlung [23]. Bei Abweichungen durch Bewegungen kann der Strahl unterbrochen und eine Korrektur vorgenommen werden.

### Ultrahypofraktionierung und intraprostatischer Boost

Die Ultrahypofraktionierung als stereotaktische Radiotherapie war am MR-Linac ohne invasive Positionsmarker in kleineren Serien jeweils gut möglich [24, 25]. Dabei wurde stets das „motion monitoring“ als Positionskontrolle während der Radiotherapie genutzt. Auch eine Planungsstudie zeigt eine ausreichende Zielvolumenabdeckung und sichere Applikation einer ultrahypofraktionierten Bestrahlung in 5 Sitzungen [26]. In einer weiteren Studie konnte gezeigt werden, dass der bis zu 30-minütige Workflow der täglichen Bildakquisition und Planadaptation vor der eigentlichen Bestrahlung bereits eine relevante Verschiebung des Zielvolumens aufgrund der Füllung und Bewegung der Beckenorgane bedeuten kann. Eine erneute Anpassung des Bestrahlungsplans an die neue Anatomie kann notwendig werden, und auch während der Bestrahlung bleibt das „motion monitoring“ wesentliche Voraussetzung für eine präzise Dosisapplikation [27].

### Outcome-Daten

Bisher liegen kaum Daten zum Outcome der Bestrahlung des Prostatakarzinoms am MR-Linac vor. Dabei ist zu berücksichtigen, das valide Ergebnisse zu onkologischen Endpunkten ein mittleres Follow-up von mehreren Jahren in der Größenordnung von mindestens 10 Jahren erreichen sollten, um sinnvoll den Stellenwert einer Behandlungsmethode zu evaluieren.

Prospektive Studien, die onkologische Outcome-Daten messen, laufen bereits, und Ergebnisse werden ab 2024 erwartet: z. B. die Beobachtungsstudien MOMENTUM (Multiple Outcome Evaluation of Radiotherapy Therapy Using the MR-linac Study, NCT 04075305) oder MIRAGE (Magnetic Resonance Imaging-Guided Stereotactic Body Radiotherapy for Prostate Cancer, NCT 04384770), die eine Ultrahypofraktionierung am MR-Linac oder CT-basiert an einem normalen Linac vergleicht.

### Toxizität

Es existieren ebenfalls wenige Ergebnisse zu Spätfolgen nach Radiotherapie am MR-Linac. Auch bezüglich der gastrointestinalen (GI) Toxizität und der urogenitalen (GU) Toxizität werden die o. g. Studien erwartet. Zur (vorübergehenden) Akuttoxizität während Therapie existieren Daten aus einer Phase-II-Studie zur Ultrahypofraktionierung. Es wurden in einem Kollektiv von 101 Patienten mit Intermediate-risk- und High-risk-Prostatakarzinom, welche jeweils 36,25 Gy in 5 Fraktionen erhielten, GU- und GI-Toxizität (RTOG, CTCAE) Grad 2 von 20 % und 3 % beschrieben; eine Toxizität Grad ≥ 3 trat nicht auf [28]. In einer weiteren Studie mit 25 Low-risk- und Intermediate-risk-Patienten, welche jeweils 35 Gy in 5 Fraktionen erhielten, wurden 12 % akute Grad-2-GU-Toxizität und keine Grad-3-Toxizität beschrieben [16]. Diese Raten decken sich mit Toxizitätsraten an normalen Linacs [5, 6].

## Zusätzliche Chancen des MR-Linac

Ausblickend sind am MR-Linac weitere Verbesserungen der Radiotherapie des Prostatakarzinoms zu erwarten.

### Funktionelle Bildgebung

Es besteht die Hoffnung, dass durch Implementierung von Informationen aus funktionellen Sequenzen in die Bestrahlungsplanung eine Therapieoptimierung erreicht werden kann. Für die Zielvolumenkonturierung, insbesondere für intraprostatische Boosts, wird dies bereits genutzt [29, 30]. Darüber hinaus bietet der MR-Linac die Option, funktionelle Bildgebung, insbesondere DWI, auch während der Therapie als prognostischen Parameter bzgl. Outcome oder Toxizitätsentstehung zu nutzen und ohne Umwege der Fusionierung bei Offline-Workflows direkt in die adaptive Bestrahlungsplanung und -applikation in Echtzeit zu integrieren sowie individuelle Konzepte wie „dose painting“ zu ermöglichen [31, 32]. Dieser Ansatz der individuell biologisch adaptierten Radiotherapie anhand von Informationen aus funktionellen Sequenzen wird in der Strahlentherapie schon länger verfolgt [33], und aktuell widmen sich viele Arbeitsgruppen diesem Thema. Erste Ergebnisse scheinen vielversprechend: DWI und dynamisch kontrastverstärkte Sequenzen können sicher und reproduzierbar durchgeführt werden [20, 34]. Bisher existiert aber noch kein klinisch validiertes Modell, das eine Vorhersage von Tumoransprechen oder Toxizität für die Radiotherapie des Prostatakarzinoms quantitativ ermöglicht.

### Reduktion der Sicherheitssäume

Durch verbesserte Abgrenzbarkeit der Organe und tägliche Planadaptation an die aktuelle Anatomie könnte der interfraktionelle Sicherheitssaum reduziert werden [35]. Daher muss untersucht werden, ob eine Reduktion der Sicherheitssäume von CTV („clinical target volume“) auf PTV („planning target volume“) erfolgen kann, ohne dass durch „target miss“ aufgrund intrafraktioneller Verschieblichkeit die Tumorkontrollrate sinkt. In einer aktuellen Simulationsstudie zur ultrahypofraktionierten Radiotherapie des Prostatakarzinoms am MR-Linac fand eine schwedische Gruppe relevante Unterdosierungen im Zielvolumen mit abnehmenden Sicherheitssäumen [27]. Es bleibt abzuwarten, ob die intrafraktionelle Verschiebung des Zielvolumens sowohl während der Planadaptation als auch während der Dauer der Bestrahlung ausreichend berücksichtigt werden kann. Perspektivisch könnten Tracking-Verfahren dieses Problem lösen [21].

### Autokonturierungs-Tools und automatische Planberechnung

Aktuell stellt die manuelle tägliche Neukonturierung den zeitaufwändigsten Arbeitsschritt am MR-Linac dar [24]. Die Implementierung von Autokonturierungstools und anderen Applikationen zur Workflow-Automatisierung und -Beschleunigung stellen (nicht nur am MR-Linac) einen Forschungsschwerpunkt in der Strahlentherapie und Radiologie dar. Diesbezüglich sind zeitnah Verkürzungen der täglichen Therapiedauer pro Patient zu erwarten [36]. In einer „Proof-of-principle“-Studie konnte bereits eine vollautomatisierte Konturierung und Bestrahlungsplanung durchgeführt und diesen Plan online-adaptiv genutzt werden [37].

### MR-only-Workflow

Die Bestrahlungsplanung anhand von MRT und daraus errechnetem synthetischem CT ist bereits seit einigen Jahren klinisch evaluiert [38]. Die Ungenauigkeiten der Dosimetrie sind bekannt und liegen generell unter 1 % [39]. Durch Verzicht auf eine Planungs-CT kann die Strahlenbelastung für Patienten reduziert werden. Bei einigen Patienten wird z. B. aufgrund von MR-Artefakten durch Prothesen allerdings auch zukünftig eine CT notwendig sein.

## Einschränkungen

Limitationen stellen die notwendige* MR-Tauglichkeit* der Patienten, die *Sitzungsdauer pro Patient *von 20–45 min und der *Ressourcenverbrauch *dar: Der Betriebsaufwand für das Team mit MTRA, Arzt und Medizinphysiker sowie QA-Anforderungen ist im Vergleich zur *Standard*-CT-gestützten Strahlentherapie erheblich. Zusätzlich besteht ein hoher Bedarf für eine enge Kooperation mit MR-Physikern und -Radiologen, damit die Vorteile der Hybrid-MR-Technologie in vollem Umfang genutzt werden können [24, 40].

## Fazit für die Praxis

- Der 1,5-T-MR-Linac kann die bestehenden Strahlentherapie-Indikationen für das Prostatakarzinom abdecken und bietet dabei konzeptionell die Vorteile des MRT-Weichteilkontrasts und der täglichen individuellen Planadaptation in Echtzeit.
- Darüber hinaus erscheinen stereotaktische Bestrahlungskonzepte einschließlich Boost intraprostatischer Tumorläsionen mit Schonung der Harnröhre am MR-Linac vielversprechend.
- Eine Live-Bildgebung während der Radiotherapie sichert die Lagerungskontrolle.
- Erste Ergebnisse zeigen eine gute Machbarkeit von konventionellen Konzepten und Ultrahypofraktionierung ohne intraprostatische Marker und vergleichbare Akuttoxizität.
- Aussagekräftige Langzeitergebnisse großer Kollektive stehen noch aus.
- Zusätzlich werden neue Therapiekonzepte verfolgt, u. a. Therapieindividualisierung und Response-Assessment anhand funktioneller MRT-Sequenzen.
- Insbesondere deshalb besteht ein hoher Bedarf an enger Kooperation zwischen allen Berufsgruppen der Radiologie, Medizinphysik und Radioonkologie.
